# Identification of Fungus GZ in Buckwheat Rhizosphere and Its Promoting Effect in Buckwheat Seed Germination

**DOI:** 10.3390/plants13233360

**Published:** 2024-11-29

**Authors:** Xiaona Zhang, Guimin Yang, Lingdi Gu, Can Liu, Qingfu Chen

**Affiliations:** 1 Research Center of Buckwheat Industry Technology, College of Life Science, Guizhou Normal University, Guiyang 550025, China; 222100100453@gznu.edu.cn (G.Y.); 194512020024@gznu.edu.cn (L.G.); 2 School of International Education, Guizhou Normal University, Guiyang 550025, China; christine53@163.com

**Keywords:** buckwheat rhizosphere fungi, isolation and identification, seed germination, *Isaria cateniannulata*

## Abstract

To obtain fungal strains that enhance plant growth in the rhizosphere soil of buckwheat, we utilized morphological and molecular biological methods to identify 10 fungal strains from the rhizosphere soil and subsequently evaluated their effects on seed germination. The results demonstrated that all 10 fungal strains were classified as *Isaria cateniannulata*. The spores of these strains significantly enhanced the germination of buckwheat seeds, with germination rates improving by 3.46% to 700.75% compared to the control group. This study fills the gap in understanding *I. cateniannulata* as soil rhizosphere fungi, providing a foundation and materials for the seed coating technology of buckwheat seeds.

## 1. Introduction

Buckwheat belongs to the Polygonaceae family, Fagopyrum genus, and is a dual-purpose grain and medicinal crop characterized by a short growth cycle and strong adaptability, including the cultivated types of *Fagopyrum esculentum* Moench, *F. tataricum* (L.) Gaertn, and *F. cymosum* (Trevir.) *Meisn.* [1]. Its seeds are rich in amino acids, dietary fiber, and flavonoids, among others [2], and have effects such as lowering blood sugar, reducing cholesterol, and enhancing immune function, as well as anti-inflammatory and antibacterial properties [3]. The germination stage of seeds is a critical period for the formation, expansion, and continuous renewal of plant populations [4]. This stage is primarily influenced by the variety’s inherent traits, such as low seed vigor [5,6,7] and poor adaptability [8], as well as external environmental factors, including diseases [9], low temperature [10], drought [11], salinity [12,13,14,15], alkalinity and heavy metal pollution [16,17], etc. To improve the germination rate of buckwheat seeds during this stage, researchers have explored various seed germination promotion technologies, among which plant root-associated microorganisms have become a focus of attention in the field of agricultural science due to their growth-promoting effects on crops and lack of toxic side effects on the environment.

Plant rhizosphere soil fungi refer to the fungi living in the rhizosphere of plants. They can be mycorrhizal fungi, saprophytic fungi, or pathogenic fungi. One type of rhizosphere microorganisms can settle in plant tissue cells and form a mutually beneficial relationship with host plants. The other type can form sheaths around the root tip and extend its network into the soil [18,19]. Currently, the obtained plant rhizosphere soil fungi mainly include *Trichoderma*, *Talaromyces*, *Fusarium*, *Phytophthora*, *Penicillium*, *Rhizoctonia*, *Gliocladium*, *Phoma*, etc. [20,21,22,23,24]. These rhizosphere soil fungi typically enhance seed vitality, promote seed germination, and break seed dormancy. For example, *Trichoderma* sp. can promote the germination and growth of seeds of Mongolian milkvetch [25]; the *Ceratobasidium* strain GS2 can effectively promote the germination of Panax notoginseng seeds [26]; four strains of fungi isolated from the rhizosphere of Tibetan barley significantly promote the germination of barley seeds [27]; *Aspergillus terreus* promotes seed germination of Dichanthium annulatum, Bothriochloa pertusa, and Setaria glauca [28]; and *Trichoderma* sp. can increase the content of soluble sugars and soluble proteins in the seed germination process of Syringa seeds by producing gibberellins and other substances, promoting seed germination [29,30]. Although plant rhizosphere soil fungi have shown a promoting effect on seed germination in various plants, whether there are fungi in the rhizosphere soil of buckwheat that promote seed germination remains to be further explored.

Therefore, this study aims to explore the effects of 10 strains of fungi GZ isolated from buckwheat rhizosphere soil on seed germination, laying the foundation for the basic research and potential application value of buckwheat rhizosphere soil fungi resources.

## 2. Materials and Methods

### 2.1. Test Culture Medium

The culture medium was prepared according to Peng et al. [31]: After sterilizing 90 mL of potato dextrose broth (PDB) and 90 mL of potato dextrose agar (PDA), the temperature was lowered to around 50 °C. Subsequently, 10 mL of a 500 µg/mL concentration of streptomycin and ampicillin, which had been filtered and sterilized with a 0.22 µm membrane filter, were added separately to achieve a final antibiotic concentration of 50 µg/mL in the culture medium [32].

### 2.2. Isolation of Test Strains

From 2019 to 2020, different healthy buckwheat plants grown in the main producing area of Guizhou were selected. The soil around the buckwheat roots was brushed with a brush, and 5 g of soil were placed in a 250 mL triangular flask with 90 mL of sterile water. The mixture was shaken in a shaker at 200 r/min for 3 h. After filtering the soil liquid with double-layer filter paper and performing gradient dilution, 200 µL of a 1 × 10^6^-fold diluted solution were spread on PDA solid medium for cultivation [33]. After 24 h, single colonies were selected for further purification. The purified strains were preserved in 30% glycerol. In this study, 10 fungi from buckwheat rhizosphere soil were selected and named GZ1 to GZ10, as shown in Table 1.

### 2.3. Identification of Test Strains

#### 2.3.1. Morphological Identification

Following the method of Peng et al. [31], the strains mentioned in Section 2.2 were placed in a constant temperature incubator at 25 ± 1 °C and 70% relative humidity for 14 days of continuous cultivation in the dark. The morphological characteristics of the strains were determined by observing their macroscopic morphology and microscopic structure. Morphological identification mainly refereed to materials from “Flora of Fungi China” [34].

#### 2.3.2. Molecular Biology Identification

Genomic DNA extraction followed the protocol of the fungal genomic DNA extraction kit (Solarbio Inc. (Beijing, China), Model D2300). PCR amplification was conducted using the universal fungal primers ITS1 (5′-TCCGTAGGTGAACCTGCGG-3′)/ITS4 and ITS4 (5′-TC CTCCGCTTATTGATATGC-3′) [35], with a reaction system of 30 μL, 2 μL DNA, 1 μL each of primers ITS1 and ITS4, 25 μL of TaqMaster Mix, and 1 μL ddH_2_O. The amplification program was as follows: initial denaturation at 98 °C for 5 min; denaturation at 98 °C for 30 s, annealing at 56 °C for 30 s, and extension at 72 °C for 30 s, for a total of 35 cycles. The final extension was at 72 °C for 5 min, and it was stored at 4 °C. The amplified products were detected by agarose gel electrophoresis and then sent to Sango Biological Engineering (Shanghai) Co., Ltd. (Shanghai, China) for sequencing. The gene sequences of the test strains were compared to the NCBI database using BLAST (https://blast.ncbi.nlm.nih.gov/Blast.cgi, accessed on 20 September 2024). Fungal sequences with more than 90% homology were downloaded from the comparison results, and a phylogenetic tree of nucleotide sequence sequences was constructed using MEGA7 [36] software, employing the neighbor-joining (NJ) method with 1000 bootstrap replicates. A bootstrap value greater than 75% indicates reliable data.

### 2.4. Impact of Test Strains on the Germination of Buckwheat Seeds

In order to achieve higher economic benefits, two main cultivated varieties (one-year-old herb) of *F. tataricum* (NO. 18) and *F. esculentum* (Hongtian NO. 1) were selected as experimental materials. A total of 30 seeds of consistent size from each variety were selected, washed with 75% alcohol for 1 min, disinfected with 1% sodium hypochlorite for 15 min, and rinsed with sterile water to a neutral pH. The seeds were then completely immersed in 15 mL of a spore suspension with a concentration of 1 × 10^6^ spores/mL for 2 h at room temperature (placed in a 5 cm × 15 cm × 15 cm germination box), with 3 biological replicates. The control group was immersed in liquid culture medium and sterile water for the same duration under identical conditions as the experimental group.

After soaking, the seeds were placed in a germination box with 5 seeds per row in 6 rows, with 3 replicates. The seeds were cultivated continuously for 3 days in a growth chamber at a temperature of 25 ± 1 °C, relative humidity of 70 ± 5%, and a light cycle of L:D = 12:12. The radicle breaking through the seed coat is used as a sign of seed germination. The number of germinated seeds was recorded daily, and the length and diameter of the sprouts were measured on the 3rd day using a vernier caliper, following the method described by Peng [31].

### 2.5. Data Processing and Analysis

The calculation formulas of germination rate and germination index are as follows:

Germination rate (%) = (number of germinated seeds/total number of seeds tested) × 100%.

Germination index (GI) = ∑(Gt/Dt), where Gt is the number of germinated seeds on day t, and Dt is the corresponding number of days for germination. Basic data analysis and plotting were conducted using Excel 2010 and Origin 2022. Statistical analysis was performed using IBM SPSS 25. Independent sample *t*-tests were used to analyze the significance of differences between two groups at the 0.05 level. Tukey’s method was applied at the 0.05 level to analyze the significance of differences among multiple groups. Image processing was conducted with Adobe Illustrator 2023.

## 3. Results and Analysis

### 3.1. Morphological Analysis of Buckwheat Rhizosphere Fungi

**Figure 1:**
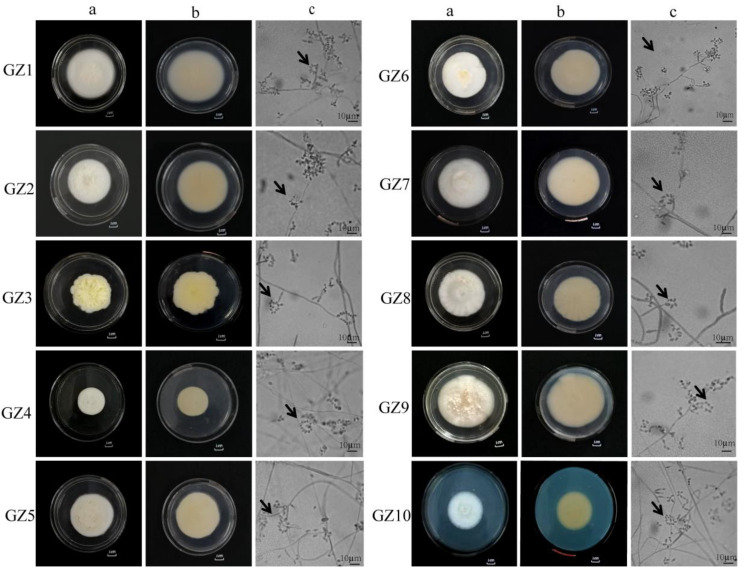
Culture characteristics and microstructure of 10 GZ strains. (**a**) represents the positive culture characteristics of the strain; (**b**) represents the reverse culture characteristics of the strains; (**c**) represents the microstructure of the strain, and the arrow in c represents the imbricate arrangement of the *I. cateniannulata*; GZ1–GZ10 represented the strain number of 10 strains.

As shown in Figure 1, the front of all strains was white, villous, flat, and dense, and some have protrusions (Figure 1—GZ3a, 1—GZ9a). The reverse side was light yellow, with some strains having folds (Figure 1—GZ1b, 1—GZ6b). These characteristics were related to the different separation sites and optimal growth conditions [12,37]. The mycelium of 10 GZ strains (see Table 2 for details) was smooth, with branches and septa. There were 2-4 phialides on the conidiophores, with the base of the phialides being spherically expanded and suddenly thinning upwards at 1/2 of the full length. The conidia were transparent and round or oval and formed imbricate conidiospore rings (Figure 1c), consistent with the description of two new species of the genus Paecilomyces discovered by Liang [38]. Based on the morphological characteristics of the cultured strains, it was preliminarily determined that the 10 GZ strains were *I. cateniannulata*.

### 3.2. Molecular Biological Analysis of Buckwheat Rhizosphere Fungus GZ

**Figure 2:**
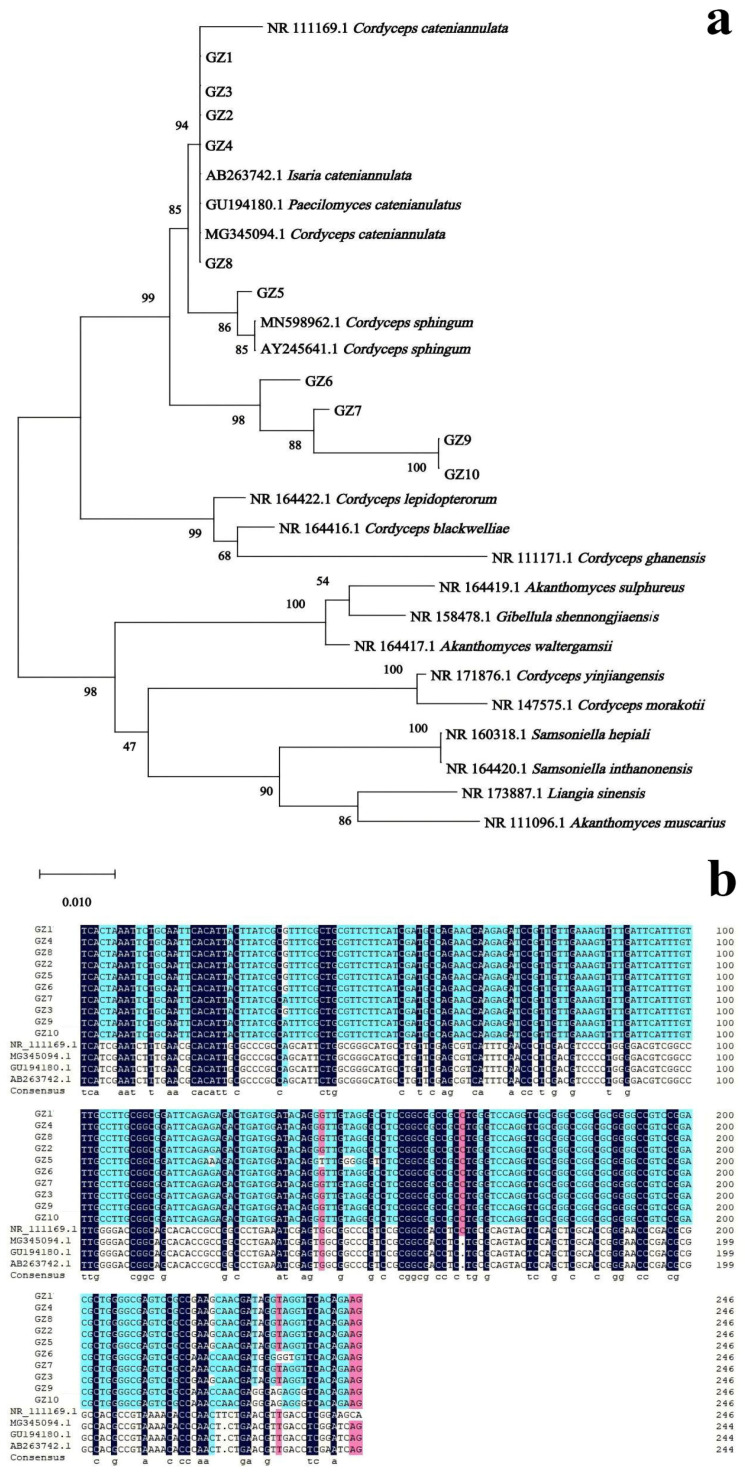
Phylogenetic tree and homologous sequence alignment of 10 strains. (**a**) represents the phylogenetic tree of 10 strains; (**b**) represents the best sequence alignment between 10 isolates and NCBI BLAST. The distance scale in the phylogenetic tree refers to the unit length of the difference value between organisms or sequences, which is equivalent to the scale of the phylogenetic tree.

The genomic DNA of each strain was extracted for PCR amplification. The PCR products obtained from 10 strains had similar sequence lengths, ranging from 500–750 bp, consistent with the size of the *I. catenianulatus* sequence. Sequencing results were based on ITS1/ITS4, a blast comparison was performed in NCBI, and a phylogenetic tree was constructed using Megalign (Figure 2a). The results showed that strains GZ1, GZ2, GZ3, GZ4, and GZ8 exhibited nucleotide sequences on the same smallest branch as *C. cateniannulata* (Genbank: NR111169.1), *I. catenianulatus* (Genbank: AB263742.1), *P. catenianulatus* (Genbank: GU194180.1), and *C. cateniannulata* (Genbank: MG345094.1) with a bootstrap value of 94%, indicating high reliability. Strains GZ5, GZ6, GZ7, GZ9, and GZ10, fiveother identified strains, along with *C. cateniannulata* (Genbank: NR111169.1), *I. cateniannulata* (Genbank: AB263742.1), *P. catenianulatus* (Genbank: GU194180.1), and *C. cateniannulata* (Genbank: MG345094.1) were clustered on a single evolutionary branch with a bootstrap value of 99%, demonstrating high reliability (Figure 2a,b). Combining morphological identification and molecular biological characteristics, the 10 strains of GZ can all be identified as *I. cateniannulata*.

### 3.3. Analysis of Promoting Buckwheat Seed Germination by I. cateniannulata

#### 3.3.1. The Influence of Spore Suspension of *I. cateniannulata* on Germination Process of Buckwheat Seeds

**Figure 3:**
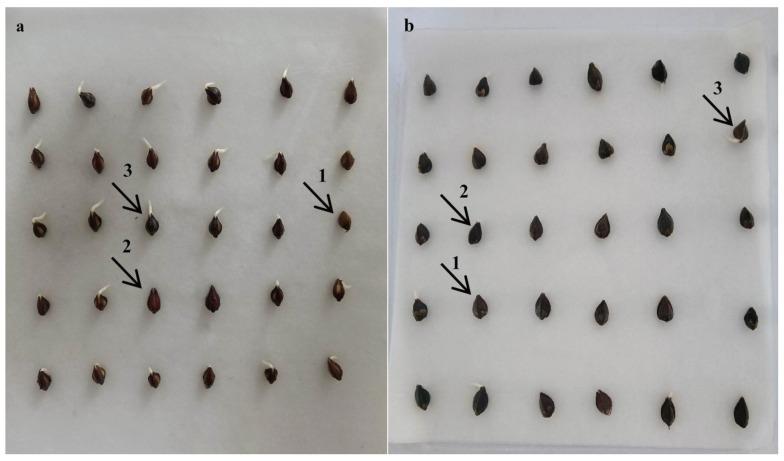
Buckwheat seed germination stage diagram. (**a**) represents the NO. 18 seeds treated with the aerial conidia of GZ1 strain for 24 h; (**b**) represents the seeds of Hongtian. treated with aerial conidia of GZ1 strain for 24 h; a1 and b1 represent the completion of imbibition period; a2 and b2 represent the completion of sprouting period; a3 and b3 represent germination period completion.

The beginning of seed germination is imbibition, and the end is the emergence of the radicle from the seed coat [39,40,41,42]. The seed germination process of *F. tataricum* and *F. esculentum* varieties in this study is shown in Figure 3. The imbibition period of *F. esculentum* seeds was 0–16 h, the sprouting period was 16–20 h, and the germination period was 20–24 h; the imbibition period of *F. tataricum* seeds was 0–12 h, the sprouting period was 12–16 h, and the germination period was 16–20 h; the imbibition period of *F. esculentum* is longer than that of *F. tataricum*. This is because *F. esculentum* needs to consume more water during the imbibition period than *F. tataricum*, and during germination, the relaxation of the seed coat cells of *F. tataricum* allows more water to penetrate [43].

#### 3.3.2. The Influence of Spore Suspension of *I. cateniannulata* on Buckwheat Seed Germination Rate

**Figure 4:**
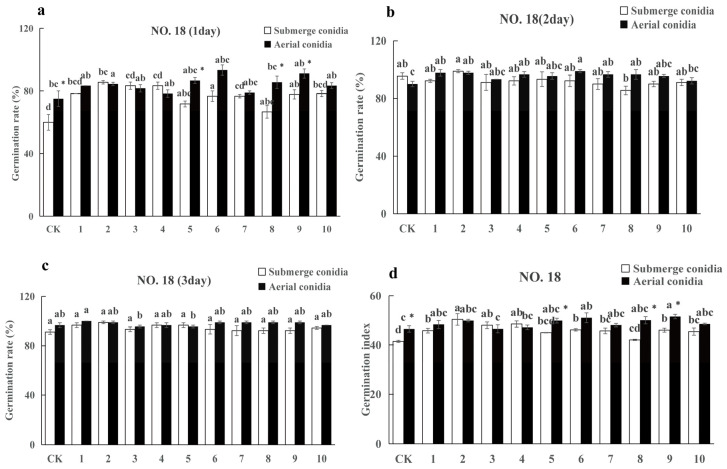
Seed soaking with spore suspension of strain GZ promoted NO. 18 seed germination. (**a**) represents the germination rate of the first day; (**b**) represents the germination rate of the second day; (**c**) represents the germination rate of the third day; (**d**) represents germination index. Note: Different lowercase letters indicate significant differences between different strains (*p* < 0.05). “*” indicates significant differences (*p* < 0.05) between the conidia and spores of the same strain on the same day.

**Figure 5:**
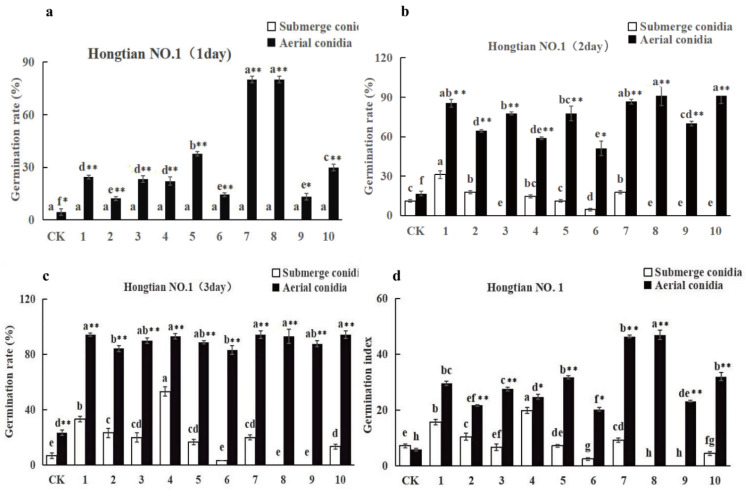
Seed soaking with spore suspension of GZ strain promoted seed germination of Hongtian NO. 1. (**a**) represents the germination rate of the first day; (**b**) represents the germination rate of the second day; (**c**) represents the germination rate of the third day; (**d**) represents germination index. Note: Different lowercase letters indicate significant differences between different strains (*p* < 0.05). “*” indicates significant differences (*p* < 0.05) between the conidia and spores of the same strain on the same day, while “**” indicates extremely significant differences (*p* < 0.01) between the conidia and spores of the same strain on the same day.

The germination rates of *F. tataricum* (Figure 4) and *F. esculentum* (Figure 5) seeds increased gradually over time when inoculated with spore suspensions and conidial suspensions of 10 strains of *I. cateniannulata*. By the third day, all parameters reached a relatively stable level (Figure 4a and Figure 5b).

On the third day, the germination rates of buckwheat seeds infected by aerial conidia were higher than those treated by spore suspension (Figure 4c and Figure 5c). The germination rates of buckwheat seeds soaked in aerial conidia suspension (except for GZ3 and GZ5) were higher than those in the control group, and the germination rates of *F. tataricum* seeds and some *F. esculentum* seeds soaked in submerged conidia suspension (except for GZ5, GZ6, GZ8, GZ9, GZ10) were higher than those in the control group. This is because the colonization of spores quickly decomposes the nutrients inside the seeds and promotes seed germination. The germination rate of the same buckwheat seed soaked by different strains varied, reflecting differences in growth-promoting ability among strains [44].

The seed germination index is an indicator of seed vitality. Inoculating different buckwheat seeds with spore suspensions of 10 fungal strains increased the germination index, consistent with the trend of germination rate (Figure 4a–d and Figure 5a–d). The germination index of aerial conidia was higher than that of submerged conidia, which is attributed to the fact that *I. cateniannulata* spores can enhance the vitality of buckwheat seeds [45]. It is speculated that *I. cateniannulata* spores indirectly affect seed vitality by influencing seed enzyme activity.

Overall, the promoting effect of *I. cateniannulata* on *F. esculentum* was greater than that on *F. tataricum*, and the effect of aerial conidia suspension was greater than that of submerged conidia suspension (Figure 4 and Figure 5). It is speculated that it is related to crop specificity, and the specific reasons need to be explored [46,47,48,49,50,51,52,53].

#### 3.3.3. The Influence of Spore Suspension of *I. cateniannulata* on Bud Length of Buckwheat Seeds

**Figure 6:**
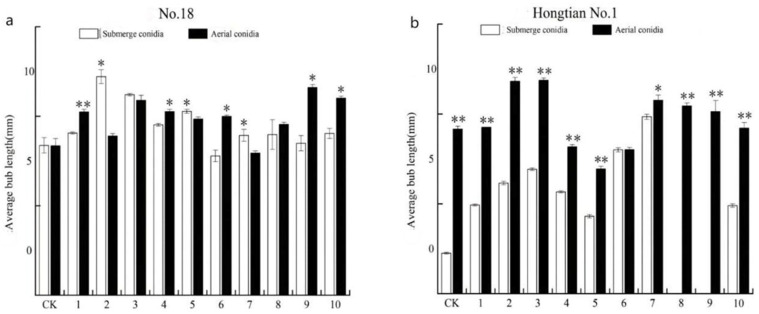
The spore suspension of GZ strain promoted the growth of buckwheat bud length. (**a**) represents the bud length of NO. 18; (**b**) stands for Hongtian NO. 1 bud length. Note: Data are presented as means ± standard error. * indicates significant difference between the control group and the experimental group (*p* < 0.05). ** indicates highly significant difference between the control group and the experimental group (*p* < 0.01).

The bud length of *F. tataricum* (Figure 6a) and *F. esculentum* (Figure 6b) increased gradually over time when soaked in the conidial suspension and spore suspension of 10 strains of *I. cateniannulata*.

On the third day, the lengths of the sprouts of buckwheat seeds soaked in aerial conidia suspensions (except for strains GZ3, 5, 6, and 7) were higher than those soaked in submerged conidia suspensions, indicating that aerial conidia had a better promoting effect on seed sprouting. Additionally, the lengths of the sprouts of buckwheat seeds soaked in aerial conidia suspensions (except for strain GZ7) were higher than those in the control group. Similarly, the lengths of the sprouts of some bitter and sweet buckwheat seeds soaked in submerged conidia (except for strains GZ6, 8, and 9) were higher than those in the control group. This suggests that both types of spores promoted the growth of buckwheat, likely through seed soaking and colonization in the seeds, potentially by increasing levels of proline, soluble total sugars, and indole-3-acetic acid as osmoprotectants, thus coordinating sprout growth [49]; however, further verification is needed.

Overall, the suspension of aerial conidia demonstrated a better effect on the promotion of bud growth. Compared with *F. tataricum*, it had a more pronounced effect on the promotion of *F. esculentum* buds.

#### 3.3.4. The Influence of Spore Suspension of *I. cateniannulata* on Bud Diameter of Buckwheat Seeds

**Figure 7:**
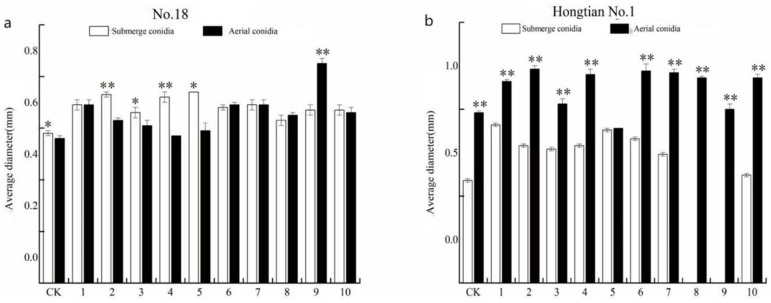
The spore suspension of GZ strain promoted the growth of buckwheat bud diameter. (**a**) represents the bud diameter of NO. 18; (**b**) stands for Hongtian NO. 1 bud diameter. * indicates significant difference between the control group and the experimental group (*p* < 0.05). ** indicates highly significant difference between the control group and the experimental group (*p* < 0.01).

The bud diameters of *F. tataricum* (Figure 7a) and *F. esculentum* (Figure 7b) increased gradually over time when soaked in the conidial suspension and spore suspension of 10 strains of *I. cateniannulata*.

On the third day, the bud diameters of buckwheat seeds soaked in aerial conidia suspensions (except strains GZ2, GZ3, GZ4, GZ5, and GZ7) were higher than those in submerged conidia suspensions, indicating a better growth-promoting effect of aerial conidia. The diameter of the seedlings soaked in conidial suspension was higher than that of the control group. Additionally, the diameter of the sprouts of some *F. tataricum* seeds and *F. esculentum* seeds soaked in submerged conidia suspensions (except for strains GZ8 and GZ9) was higher than that of the control group, indicating that both types of spores had a promoting effect on the diameter of buckwheat sprouts. This effect may be due to the production of plant growth hormones by the spores, which could enhance cell division and elongation, thereby increasing the sprout diameter [53]. However, the specific mechanism still needs further investigation.

Overall, the effect of aerial conidia suspension on bud diameter growth was better. Compared with *F. tataricum*, the effect of aerial conidia suspension on bud diameter growth of *F. esculentum* was more pronounced.

## 4. Discussion and Conclusions

Currently, plant rhizosphere soil fungi are mainly obtained from the plant root surface and surrounding soil, playing crucial roles in decomposing organic matter, participating in nutrient cycling and exchange, promoting plant growth, and improving soil productivity [54,55]. In this study, 10 fungi were isolated from buckwheat rhizosphere soil, all of which belong to *I. cateniannulata*. In previous studies, *I. cateniannulata* were mostly isolated from various entomopathogenic fungi, including larvae of *Allantus luctifer* [31], pupae of the *Homona coffearia Nietner* and the *Tenodera sinensis* [56], the cocoons of the *Adoxophyes honmai Yasuda*, and the adult of the Coleoptera [45], as well as the pupae of Lepidoptera [57,58]. This study is the first to isolate and classify them as rhizosphere soil fungi, similar to the results obtained by Niu [59], who isolated *Metarhizium anisopliae* and *Beauveria bassiana* from soil and classified them as soil fungi.

There were differences in the morphological characteristics of different *I. cateniannulata* strains, and they showed a high degree of diversity. In this study, differences were found among different strains of *I. cateniannulata* isolated from soil in different buckwheat planting areas. This is consistent with the results of Han [60], Zhu [61,62], and others who [63,64] found characteristic differences among strains of *I. cateniannulata* collected from different regions. Moreover, Wang et al. [65,66] found a high degree of similarity among *B. bassiana* strains from the same geographical source, which also indirectly reflects the phenotypic differences among strains from different regions. However, the specific mechanisms underlying this phenomenon require further investigation.

Crop rhizosphere soil fungi play a significant role in plant health by colonizing the plant rhizosphere and enhancing nutrients and water uptake [67], thus promoting seed germination and plant growth [68]. Zhao et al. [27] identified fungal strains from rhizosphere soil fungi with phosphorus solubilization and nitrogen fixation capabilities, which had different promotion effects on barley seed germination. Gillespie-Sasse found that rhizosphere soil fungi could produce gibberellic acid (GA), which regulates seed germination as well as root and shoot development [69]. Yao [70] found that 15 fungal liquids significantly promoted the seed germination speed of plants, mainly related to the production of protease by the strains. Peng et al. [71] observed that soaking buckwheat seeds in *I. cateniannulata* led to a significant increase in seed germination, likely due to its influence on the release of starch in endosperm and protein in the embryo [72]. Shi et al. [73] noted that fungi colonized in seeds penetrate the outer seed coat through hyphae, invade between the seed coat and the embryo, elongate the embryo radially, differentiate roots, and are covered by hyphae, and in the late stage of embryo development, hyphae invade the outer tangential wall of the epidermis for nutrient exchange, promoting seed germination. In this study, all 10 strains were shown to promote buckwheat seed germination. However, further investigation is needed to clarify whether this promotion is related to specific mechanisms such as nitrogen fixation, phosphorus solubilization, auxin secretion, enhanced nutrient exchange, etc.

*I. cateniannulata* has been shown to promote crop growth and enhance seed vigor, though the effects vary among different fungal strains and spore suspensions on different crop varieties. Qayyum MA et al. [74] found that two strains of *B. bassiana* promoted tomato growth, while one strain showed the side effect of reducing aboveground dry weight and fruit weight. Wang et al. [75] investigated two strains of *M. anisopliae*; one strain effectively colonized rice roots, enhancing seed germination and various physiological indicators such as plant height, root length, fresh weight, dry weight, and chlorophyll content, while the other strain had no significant effect. Similarly, Liao et al. [76] found that different strains of *M. anisopliae* varied in their growth-promoting effects on crops, although none affected seed germination. Peng et al. [31] studied eight strains of *I. cateniannulata* and found that they improved the germination rate, germination index, shoot length, and shoot diameter of six kinds of buckwheat seeds to varying extents. These findings are similar to the results of this study, with most strains of conidia having a good promoting effect on germination rate, germination index, and shoot diameter, further indicating that different *I. cateniannulata* have different effects. While colonizing buckwheat seeds, the *I. cateniannulata* also increased the seed SOD, POD, and CAT enzyme activities and decreased the malondialdehyde content [71]. Related studies have also shown the correlation between seed vigor and enzyme activity [50,77,78,79], suggesting that seed vigor can be improved by affecting enzyme activity [50,56], with different strains leading to different effects. Furthermore, the differences in germination rate and germination index between the two seeds in this study are attributed to seed specificity and seed coat permeability [80]. The different effects of the same strain on different seeds are related to the thickness of seed coat, type and behavior of seed coat cracking [51], binding capacity of seed contents [48], species, growth period [46,50], and active substances promoting seed germination in the seed coat [47]. In this study, this difference is attributed to the variations in carbon sources on the cell walls of aerial conidia, hosts, and spores, resulting in different hydrophobicity, adhesion abilities to surfaces, and sensitivities to glycosidase, protease, pH values, and competition for carbohydrates [81,82]. Typically, the infection process is initiated by spore attachment to the host cell wall and invasion in hyphal form [83], which can better induce plant resistance. Aerial conidia, the primary means of asexual reproduction for the I. cateniannulata, grow on conidial stalks differentiated from the nutritionally grown mycelium. Aerial conidia have a stronger colonization ability in plant tissues. Therefore, it is speculated that I. cateniannulata aerial conidia can colonize better in plants and affect their growth-promotion effect [84].

This study first isolated 10 strains of *I. cateniannulata* from the rhizosphere soil of buckwheat and proved that their spore suspensions can increase the germination rate, germination index, and shoot length of buckwheat. However, the effectiveness of different spore suspensions varies among strains and buckwheat varieties. These findings provide a foundational basis for further development and application of *I. cateniannulata* as a germination promoter for buckwheat seeds.

## Figures and Tables

**Table 1:** Information about the strains.

Strain	Strain ID	Source Habitat	Collection Date
1	GZ1	Duyun, Guizhou	31 October 2019
2	GZ2	Weining, Guizhou	13 September 2020
3	GZ3	Duyun, Guizhou	31 October 2019
4	GZ4	Kaiyang, Guizhou	4 December 2020
5	GZ5	Duyun, Guizhou	24 October 2019
6	GZ6	Kaiyang, Guizhou	6 December 2020
7	GZ7	Kaiyang, Guizhou	6 December 2020
8	GZ8	Duyun, Guizhou	31 October 2019
9	GZ9	Weining, Guizhou	12 September 2020
10	GZ10	Duyun, Guizhou	31 October 2019

**Table 2:** Macroscopic culture characteristics of strain.

Strain	Morphological Characteristics
GZ1	The colony diameter was 70.00 ± 1.00 mm, with both sides being white. The mycelium was fluffy, and the entire colony was dense with wrinkles on the reverse side. The hyphae were smooth, branched, and septate, with a diameter of 2.3–5.7 µm. The conidiophore was 14–23 µm long, bearing 2–4 phialides, with the base of the phialides exhibiting spherical expansion. Conidia were hyaline, round, oval, or ovoid, 3.8–10.9 × 3.2–6.2 µm in size, and produced in imbricate chains (Figure 1—GZ1a, b, c).
GZ2	The colony diameter was 60.66 ± 1.52 mm. The front side was white, and the reverse side was pale yellow. The mycelium was fluffy, with the whole colony was being dense. The reverse side of the colony was circular, with a flat edge, a raised center, and a neat margin. Yellow droplets were present in the center. The hyphae were smooth, branched, and septate, with a diameter of 1.8–3.4 µm. The conidiophore was produced in 2 whorls, bearing phialides. The base of the phialides was spherical and expanded. The conidia were unicellular, hyaline, and oval to nearly spherical, measuring 5.4–9.1 × 2.1–5.6 µm in size, and usually produced in imbricate chains (Figure 1—GZ2a, b, c).
GZ3	The colony diameter was 57.00 ± 2.00 mm, with both sides of the colony being white and containing light yellow pigments. The colony was white and fluffy, with no fascicled hyphae. The colony was flat, with a fluffy edge. The hyphae were smooth, branched, and septate, with a diameter of 1.2–3.1 µm. The sporulation axis was 14.3–15.3 µm long and 1.2–1.4 µm wide, usually produced in 2 whorls, with phialides typically in 2-4 whorls. The phialides were long, awl-shaped, with slimy heads at the apex. Conidia were 2.6–6.9 × 1.1–1.6 µm in size, round or oval, and produced in imbricate chains (Figure 1—GZ3a, b, c).
GZ4	The colony diameter was 51.33 ± 2.23 mm, with the front side being white and the reverse side light yellow. The colony was fluffy, with dense mycelium and a raised center. The hyphae were hyaline, smooth, branched, and septate, with a diameter of 1.8–4.2 µm. The conidiophore was 12.6–14.4 µm long. The conidia were hyaline, round or oval, 3.8–7.6 × 3.2–6.3 µm in size, and produced in imbricate chains (Figure 1—GZ4a, b, c).
GZ5	The colony diameter was 59.33 ± 3.51 mm, with both sides of the colony light yellow, and the lower part of the front side was white and fluffy. The upper part was light yellow and branched, with a raised center and flat edges. The colony’s edge was petal-shaped and fluffy. The hyphae were 2.1–3.9 µm in diameter, septate, hyaline, and smooth. The conidiophores were short or absent, about 15.3–13.6 µm long and 1.8–1.6 µm wide. The conidium were 2.1–3.9 µm × 2.2–4.3 µm in size, hyaline, smooth, oval or nearly spherical, and produced in imbricate chains (Figure 1—GZ5a, b, c).
GZ6	The colony diameter was 64.00 ± 3.00 mm, with the front side being white and the reverse side light yellow. Yellow pigments were secreted. The hyphae were cottony, with radial grooves on the surface and a raised center, while the colony’s edge was petal-shaped. The hyphae were 2.1–4.2 µm in diameter, septate, hyaline, and smooth. The conidiophores were short, about 24.5–27.4 µm long and 2.3–2.6 µm wide, bearing 2–4 phialides. Most phialides had a base that was spherical and swollen, abruptly tapering at the upper half. The conidia were 3.9–6.1 × 2.2–4.3 µm in size, hyaline, smooth, mostly oval or nearly spherical, and produced in imbricate chains (Figure 1—GZ6a, b, c).
GZ7	The colony diameter was 56.00 ± 2.00 mm, with both sides of the colony being white and fluffy. The hyphae were hyaline, septate, and branched, with a diameter of 1.8–4.7 µm. The conidiophores were 12.1–16.3 µm long, bearing 2–4 phialides with a spherical and swollen base. The conidia were hyaline, mostly round, oval or ovoid, and produced in imbricate chains (Figure 1—GZ7a, b, c).
GZ8	The colony diameter was 68.00 ± 2.00 mm, with the front side being white and the reverse side light yellow. The colony was downy to fluffy, with dense mycelium and fascicled hyphae. The surface of the hyphae was wrinkled, with an irregularly raised center and flat edges. The mycelium was hyaline, septate, and 2.3–3.8 µm in diameter. The conidiogenous cells were long phialide-shaped, 9.0–26.0 µm long, and arose directly from the hyphae or from the conidiophores. The conidia were oval or ovoid, unicellular, hyaline, and 4.3–7.8 µm × 2.1–3.7 µm in size, commonly produced in imbricate chains (Figure 1—GZ8a, b, c).
GZ9	The colony diameter was 59.33 ± 3.05 mm, with both sides of the colony being white and containing light yellow pigments. The colony was white and fluffy, flat, and with a fluffy edge. The mycelium was hyaline, septate, branched, and 1.34–3.43 µm in diameter. The conidiogenous cells were long phialide-shaped, 12.2–24 µm long, arising directly from the hyphae or from the conidiophores. The conidia were oval or round, unicellular, hyaline, and 1.4–5.1 × 2.4–3.5 µm in size, commonly produced in imbricate chains (Figure 1—GZ9a, b, c).
GZ10	The colony diameter was 43.33 ± 2.51 mm, with the colony being white, fluffy, and flat and showing indistinct concentric rings. The front side was white, while the reverse side was light yellow, with a rounded, fluffy edge. The hyphae were hyaline, smooth, septate, and branched, with a diameter of 0.8–1.7 µm. The conidiophores were 7.9–29.6 µm long. The conidia were unicellular, hyaline, smooth, mostly oval or ovoid, measuring 2.4–4.3 × 1.9–2.9 µm, and commonly produced in imbricate chains (Figure 1—GZ10a, b, c).

## Data Availability

The original contributions presented in the study are included in the article, further inquiries can be directed to the corresponding author and the first author.
